# Evaluating Residual Stress in Carbon Fiber-Reinforced Polymer (CFRP) at Microscale Using Fiber Push-Out Experiment and Finite Element Modeling

**DOI:** 10.3390/polym15122596

**Published:** 2023-06-07

**Authors:** Quy Tung Linh Vu, Guillaume Seon, Sarvenaz Ghaffari, Andrew Makeev, Frédéric Lachaud, Miguel Charlotte, Yves Gourinat

**Affiliations:** 1 Department of Mechanical and Aerospace Engineering, The University of Texas at Arlington (UTA), 701 S. Nedderman Drive, Arlington, TX 76019, USA; tunglinh.vuquy@mavs.uta.edu (Q.T.L.V.); seon@uta.edu (G.S.); sarvi.ghaffari@uta.edu (S.G.); 2 Institut Clément Ader (ICA), DMSM, ISAE-SUPAERO, 10 Av. Edouard Belin, 31400 Toulouse, France; frederic.lachaud@isae-supaero.fr (F.L.); miguel.charlotte@isae-supaero.fr (M.C.); yves.gourinat@isae-supaero.fr (Y.G.)

**Keywords:** residual stress, micromodels, finite element method updating, fiber push-out

## Abstract

Microscale residual stress may develop during the manufacturing of Carbon Fiber-Reinforced Polymer (CFRP) composites and negatively affect apparent macroscale mechanical properties. Accordingly, accurately capturing residual stress may be essential in computational methods used for composite material design. This work presents a new data-driven methodology for the evaluation of microscale residual stress in CFRPs using fiber push-out experiments with in situ scanning electron microscopy (SEM) imaging. SEM images reveal significant through-thickness matrix sink-in deformation in resin-rich areas after nearby fibers are pushed out, which is attributed to the release of microscale process-induced residual stress. The sink-in deformation is measured experimentally, and a Finite Element Model Updating (FEMU) method is used to retrieve the associated residual stress. The finite element (FE) analysis includes simulation of the curing process, test sample machining, and fiber push-out experiment. Significant out-of-plane matrix deformation larger than 1% of the specimen thickness is reported and associated with a high level of residual stress in resin-rich areas. This work emphasizes the importance of in situ data-driven characterization for integrated computational materials engineering (ICME) and material design.

## 1. Introduction

Fiber-reinforced composite materials have been increasingly used in the design of high-performance parts for many application fields, including sports [1,2], energy [3,4], transportation [5,6], aerospace [7,8,9,10], and civil engineering [11,12,13,14,15], due to their superior mechanical, durability, and weight properties. With recent advances in computers and simulation software, there has been an increased interest in using computational material engineering to accelerate the design and qualification of composite materials used in aircraft applications, including CFRPs. At the microscale, computational models enable the representation of the anisotropic and heterogenous nature of CFRPs with high fidelity as well as the incorporation of elaborate material laws, which allow capturing the physics associated with the multiple inherent failure modes. Common applications of micromodels include homogenization and prediction of macroscale constitutive properties [16,17,18,19,20,21], evaluation of the effects of microscale irregularities on macroscale properties [22,23], and numerical analysis to support experimental measurement of microscale properties [24,25,26].

Because both fiber and matrix phases are represented explicitly, computational analysis at the microscale is also suitable for the prediction of residual stress that may develop during the curing process due to property mismatch between fibers and matrix, including thermal and cure properties. For instance, the chemical shrinkage strain of epoxy resins used in CFRPs is of the order of several percent [27,28,29], while carbon fibers are typically inert at the curing temperature [30], and their chemical shrinkage is comparatively negligible. During the cooldown phase of the curing process, additional residual stress might also build up due to the difference in the Coefficient of Thermal Expansion (CTE) between fiber and matrix. The CTE of common epoxies is around 50 ppm/°C, while the CTE of common carbon fibers used in aerospace CFRPs application is typically negative and two orders of magnitude smaller. The thermal and/or chemical microscale residual stress associated with these property mismatches can negatively affect apparent macroscale mechanical properties [31,32,33,34,35]. Accordingly, capturing residual stress in numerical micromodels may be essential for the accurate prediction of CFRP properties using computational methods.

Residual stresses are typically accounted for by performing a cure process analysis prior to the mechanical analysis used for homogenization or for supporting the characterization of microscale properties [31,32,33,34,35]. However, key properties involved in residual stress build-up at the microscale, including parameters controlling chemical shrinkage, are difficult to measure experimentally. In particular, the strong coupling of chemical shrinkage with thermal expansion combined with the dependency of the mechanical properties of epoxy on both temperature and degree of cure contributes to the complexity of characterizing chemical shrinkage and capturing its effects on residual stress. Additional challenges are related to the effects of the size of the unit cell micromodels and prescribed boundary conditions used to capture residual stresses. At the minimum, reliable experimental methods are needed for the evaluation and verification of the residual stresses predicted using micromodels.

While many experimental techniques have been proposed for the measurement of residual stress/strain in polymer matrix composites at the ply length scale [36], very few methods are currently available for residual stress measurement at the microscale. This is in part due to the length scale involved. Commonly used experimental procedures for the measurement of residual stress are destructive methods that rely on measuring deformation due to the release of residual stress upon material removal. Microscale residual stresses in CFRPs arise due to interactions between fibers and matrix during cure, which implies a governing length scale smaller than one fiber diameter. With typical carbon fiber diameters ranging from 5 to 7 μm, special equipment is needed to machine test specimens, remove material, and measure the deformation associated with the microstress release. It is worth noting that microscale residual stress/strain in CFRPs may also be strongly affected by local material irregularities, such as non-uniform fiber distribution, and vary spatially, which adds another layer of complexity to their evaluation.

Nondestructive methods for direct assessment of residual stress/strain could potentially overcome some of the challenges related to material removal and measurement of deformation in destructive techniques at the microscale. For example, the Raman spectroscopy technique is a nondestructive method that quantifies the vibration modes of molecules with an applicable resolution (0.1–1 μm) that has been used in [37,38,39,40] for evaluation of microscale residual stress in materials with crystalline microstructure. However, a poor resolution was found when the method was applied to amorphous materials [41,42,43], which includes the thermoset epoxy resins used in CFRPs. X-ray diffraction is another non-destructive method worth mentioning for the measurement of residual strains at the microscale, but the technique also requires a crystalline material microstructure [44]. Predecki et al. [45,46] proposed a solution for residual stress measurements in amorphous materials, such as epoxy resins, by dispersing crystalline microparticles into the matrix material and measuring the X-ray diffraction associated with the embedded particles. However, the complex interaction between the different materials as well as the possible effects of the particle geometry and their interface properties, make the correlation between diffraction attributes and residual strain within the epoxy difficult to quantify and validate.

The objective of this work is to address the lack of methods available for the characterization of microscale residual stress in CFRPs. Recent advances in in situ SEM with integrated nanomechanical and micromechanical test systems, allowing for high-resolution dynamic imaging while testing, are increasing our confidence in the ability to break through the current length scale limits in material characterization. In particular, this work presents a new data-driven methodology for the evaluation of microscale residual stress in CFRPs based on a fiber push-out experiment using in situ SEM. The in situ SEM-based experiment reveals significant through-thickness matrix sink-in deformation in resin-rich areas after nearby fibers are pushed out, which is attributed to the release of process-induced microscale residual stresses. To characterize and reproduce the residual stress field, an FEMU procedure is proposed. SEM images of the test samples are used to generate high-fidelity microscale finite element (FE) models capturing local fiber distribution. The FE analysis includes a simulation of the curing process, test sample machining, and fiber push-out to capture both the build-up and release of residual stress. An FEMU algorithm is used to optimize the properties associated with residual stress build-up, such as the error between the simulated matrix through-thickness deformation after fiber push-out and test measurements are minimized. After the convergence of the algorithm, the residual stress can be evaluated in the FE model. Since the fiber/matrix interface is broken during the experiment to release residual stress and measure the associated deformation, this method may be classified as destructive.

The methodology is demonstrated for a carbon-fiber/epoxy–matrix HS40/F3G material system representative of CFRPs used in aerospace applications. Results suggest high concentrations of the process-induced residual microstresses in the matrix around five-micron-diameter carbon fibers.

## 2. Methodology

**Figure 1:**
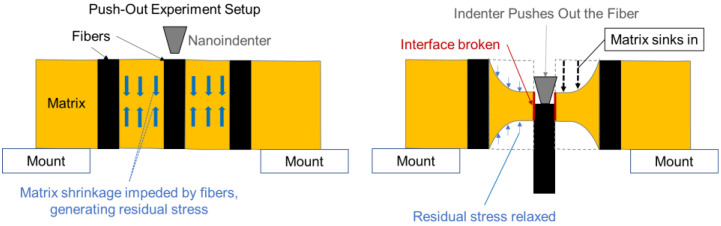
Illustration of fiber push-out experiment. Broken fiber–matrix interface partially relaxes residual stress in nearby matrix.

**Figure 2:**
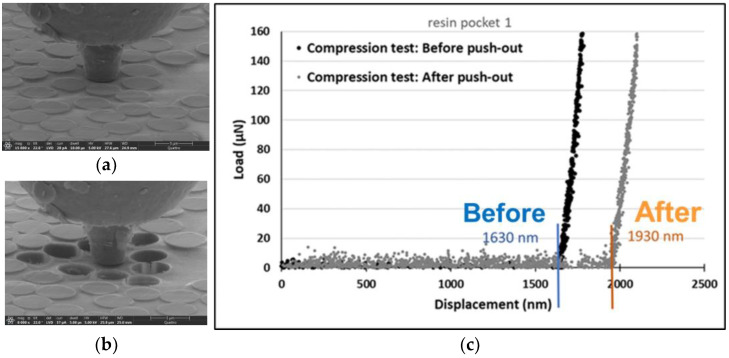
Nanoindenter measuring matrix sink-in: (**a**) before fiber push-out; (**b**) after fiber push-out; (**c**) load-displacement data showing local matrix sink-in after push-out experiment.

Originally, the fiber push-out experiment with in situ SEM was developed in Reference [25] for assessing fiber–matrix interface shear strength in CFRPs. In the experiment, a nano-indenter load frame is used to push out carbon fibers in thin membrane test samples. Recording of the maximal load allows assessment of the apparent maximum interfacial shear strength. Live SEM in situ imaging is used to ensure that the indenter probe is correctly positioned and that a consistent failure mode by interface debonding is obtained. The imaging showed significant through-thickness matrix sink-in deformation in resin-rich areas after nearby fibers are pushed out, which is attributed to the release of process-induced microscale residual stresses. This is illustrated schematically in Figure 1. The overall sink-in deformation increases as multiple neighboring fibers are pushed out. Figure 2a,b shows an example of sink-in deformation visible in the SEM image of the test sample after pushing out nine fibers.

The sink-in deformation is measured using a nanoindenter to probe the resin-rich region within the push-out fibers and measure the difference in absolute position at a reference point prior to and after push-out, as shown in Figure 2a,b. The absolute position is determined in the load-displacement response as the probe displacement when the load reading starts to pick up after initial contact (Figure 2c).

**Figure 3:**
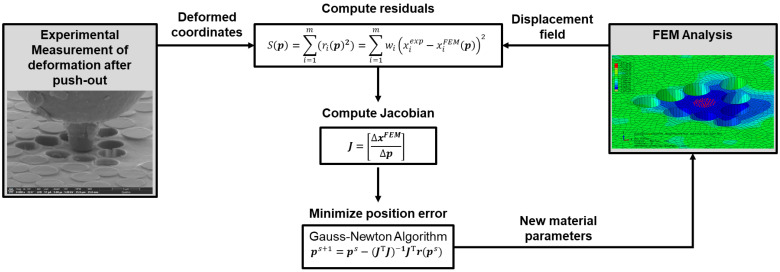
Flowchart of FEMU inverse algorithm calculating material parameters.

An FEMU procedure is proposed for the inverse characterization of the residual stress associated with the sink-in deformation measured after fiber push-out. Figure 3 illustrates the inverse algorithm process. In this study, the FEMU method is used to optimize the epoxy matrix chemical shrinkage property, such as the error between the simulated matrix sink-in deformation and the deformation measured in the test is minimized. The epoxy chemical shrinkage is selected as the optimizing variable for several reasons: chemical shrinkage is known to be a significant contributor to residual stress formation; chemical shrinkage is difficult to measure experimentally, especially at the microscale, where local random variations might occur. The inverse algorithm is written and implemented using Abaqus/CAE built-in Python scripting interface.

The FEMU procedure uses a Gauss–Newton optimization algorithm with a Jacobian matrix calculated using the finite difference method. Via iterating epoxy chemical shrinkage $\varepsilon_{0}^{chem}$, the algorithm seeks to minimize the difference between the numerically calculated matrix sink-in ($Sinkin^{FEM}$) and the experimentally measured matrix sink-in ($Sinkin^{exp}$):

(1) $$\Delta^{s}=\left|Sinkin^{FEM}-Sinkin^{exp}\right|.$$

The iterative procedure is stopped when a convergence threshold of $\Delta^{s}<1\;nm$ is reached, which corresponds to a relative error of about 0.3% of the average maximum matrix sink-in deformation measured in the test (see Section 5.1). Convergence was typically achieved in two or three iterations.

## 3. Experimental Procedure

All the experiments discussed in this study were carried out on HS40/F3G composites. HS40 is Mitsubishi (Tokyo, Japan) 12K-filament-count-tow Polyacrylonitrile (PAN)-based high modulus carbon fiber with a diameter of 5 µm. The resin system is Patz Materials and Technologies (PMT, Benicia, CA, USA) F3G, 121 °C (250 °F) curing Nanosilica-toughened epoxy (40% Nanosilica by resin weight). A thin sample for the push-out test is obtained by cutting a 5×5×5 mm piece from the unidirectional composite panel using a high-speed diamond saw. The surface perpendicular to the fibers is polished with a sequence of silicon carbide abrasive papers of 320, 600, 800, and 1200 grit. Then, the polished section is mounted on a disk grinder (Model 623, Gatan, Pleasanton, CA, USA) using a Christal bond adhesive, and the opposite surface of the sample is thinned out by lapping/polishing following the same sequence of abrasive papers until the final sample thickness is in the range of 20–30 µm. To ensure a quality finish, 0.04 µm colloidal silica suspension is used for the final polishing. Finally, a 200–300 µm wide slice was cut from the polished sample, and its thickness was measured at both edges widthwise using optical microscopy. This step was repeated until the thickness at both edges across the width was measured to be the same, which indicated the absence of the taper. These measurements were confirmed by SEM as well.

**Figure 4:**
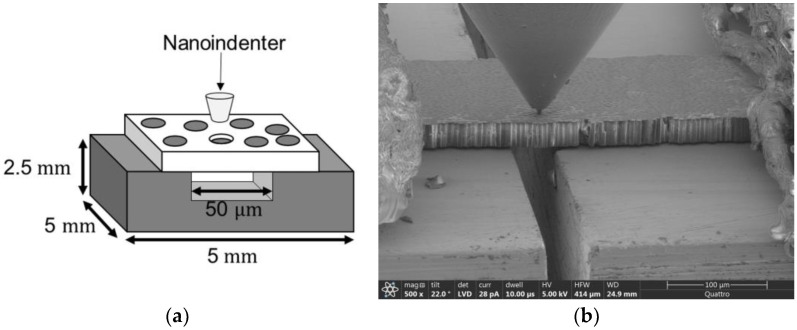
(**a**) Schematic of fiber push-out experiment. (**b**) Push-out specimen in SEM.

Next, the specimen is placed on a steel fixture with a 50 μm-wide groove engraved on its surface, and the outer part of the sample is taped down to the fixture using conductive tape. Then, the fixture with a mounted specimen is placed on a sample holder of the Bruker (Billerica, MA, USA) PI-88 PicoIndenter micromechanical load frame in the SEM. A three-plate capacitive transducer technology in the system provides high sensitivity for force measurement, and a piezo-based flexure controls displacement measurement and actuation. A flat-end diamond indenter tip with a 4 μm diameter is used to perform indentation tests. Figure 4 shows the schematic of the push-out experiment and the push-out sample in the SEM.

First, the probe is placed above the resin-rich area, and its location is saved as a reference point. Next, an indentation test is performed on the resin pocket to measure the vertical distance between the reference point and the matrix surface. This test is conducted under displacement control mode with a loading rate of 30 nm/s. This distance determines the primary location of the matrix with respect to the probe. Then, a group of fibers surrounding the matrix-rich area are pushed out of the sample (Figure 2a,b). To measure matrix deformation/sink-in after the push-out experiment, the probe is moved back to the previously saved reference point, and a new indentation test is conducted on the same resin pocket. A precise loading point is confirmed by SEM observations, and if the loading point on the matrix does not perfectly match the initial indentation test, such test results are rejected. The vertical coordinate of the matrix area is identified by a sudden change of slope in the load-displacement curves. When the probe reaches the surface of the sample and contact between tip and specimen is formed, it starts to apply load on the matrix, which appears as a load rise in the graph (Figure 2c). Therefore, matrix sink-in is obtained by comparing the recorded vertical coordinates before and after the fiber push-out test (Figure 2c). Using this method, sink-in measurement resolution is expected to be smaller (better) than 10 nanometers.

## 4. Finite Element Model

### 4.1. Mesh Generation

The finite element method (FEM) and FEMU studies in this paper are performed using Abaqus FEM simulation software, version 2022.

**Figure 5:**
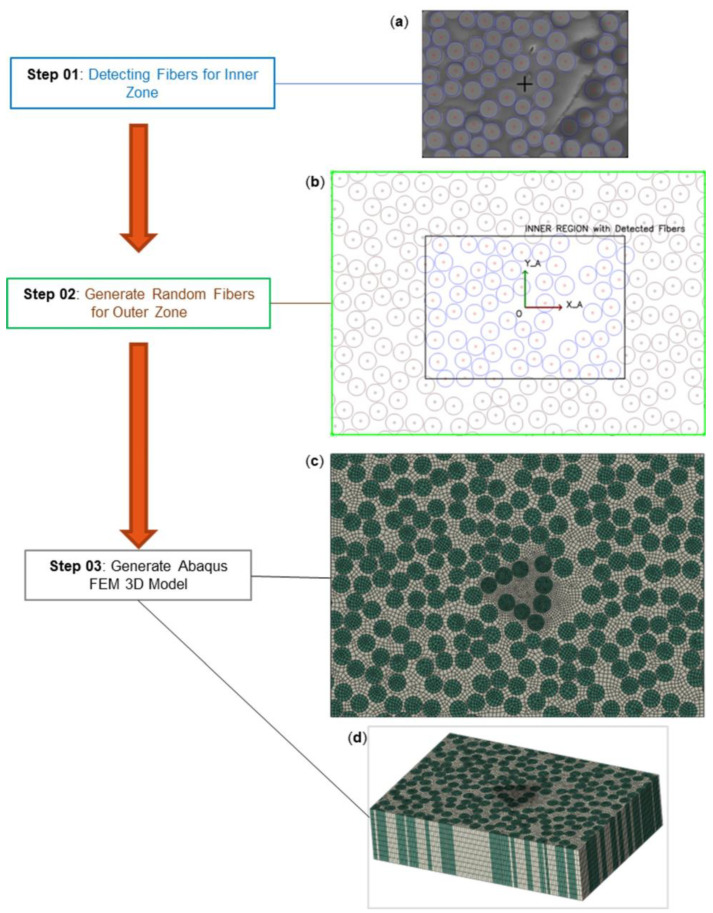
Process of creating 3D FE Model based on microscopy data. (**a**) Detected fibers in blue. (**b**) Generated fibers in brown and inner zone boundary in black. (**c**) 3D FE Model, top view. (**d**) 3D FE Model, oblique view.

FE model geometry is based on microscopy data. Microscopy images of push-out specimens provide information on in-plane fiber geometry and distribution and membrane thickness. The process of obtaining specimen geometry and building a matching FE model consists of three Steps that are illustrated in Figure 5.

In Step 1, fibers are detected from 2D microscopy images using the Hough Circle Transform algorithm that is available in the OpenCV computer vision library of the Python programming language. The fiber cross-section is assumed to be circular for the detection. The fiber detection algorithm is applied to the inner zone with push-out fibers at its center. A few undetected fibers are added manually to the result. Overlapping or too-close circle pairs have their radii proportionally adjusted so that their minimum distance is $0.08\;R_{f}$, where $R_{f}=2.45\;\mu m$ is the typical radius of HM carbon fibers. This adjustment allows a more robust meshing of the resulting FE model. Figure 5a shows the inner zone with the final fiber geometry resulting from Step 1.

In Step 2, fibers in the outer zone (outside the fiber detection area) are generated as circles of random distribution and of uniform radius $R_{f}$. All fibers in the inner and outer zones also have a minimum separation of $0.08\;R_{f}$. The outer zone fibers are generated using an Improved Random Search Algorithm (IRSA) that is based on the common Random Search Algorithm (RSA). The classical RSA suffers from the “jamming” problem that limits the resulting volume fraction to less than 50%, compared to the fiber volume fraction of >60% found in aerospace composites. To solve the jamming problem, at each iteration, the IRSA loops through every generated circle and pulls each circle toward its closest neighbors by random distances, creating room for new fibers to be generated. In this paper, the objective volume fraction for the IRSA is 54%.

The procedure of extracting specimen geometry from microscopy images requires several assumptions and simplifications: the membrane surface is perfectly flat and parallel to the image plane; the microscopy image suffers no aberration or distortion. With the scaling ratio provided by the microscope system, a projection can be defined to transform specimen geometry from the image into the real in-plane coordinates system that would be used to generate the FE model.

In Step 3, with specimen geometry from Step 1 and Step 2, a 2D surface geometry is generated in Abaqus. This 2D geometry is meshed by the Abaqus default meshing tool (Figure 5c). The 3D model geometry is then created by extruding the 2D geometry by the membrane specimen thickness (Figure 5d), assuming the thickness is uniform. The reference FE model in this study utilizes the geometry shown in Figure 5.

Mesh and size convergences have been verified in such a way that the effects of significant changes in mesh density and model size on FEM-computed sink-in deformation are negligible. For more details on these convergence studies, please refer to Appendix A.

### 4.2. Analysis Steps

**Figure 6:**
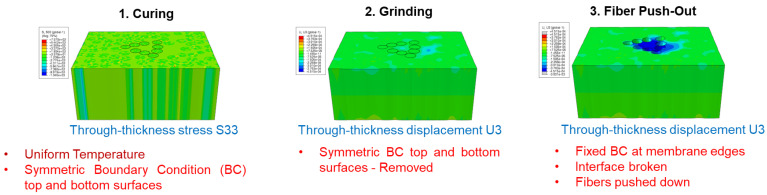
Example of one FEM simulation. Epoxy-curing shrinkage generates through-thickness stress. Through-thickness membrane sink-in deformation is clearly shown after fiber push-out.

The FEM simulation replicates three processes, as illustrated in Figure 6: specimen curing; specimen grinding (into a thin membrane); and a push-out experiment. The analysis is performed using Abaqus Fully Coupled Temperature-Displacement solver.

During curing and all the following stages of the simulation, a uniform temperature field is applied to the model. Applied temperature over-simulated time follows the F3G epoxy curing cycle, as described later in this section. To verify the validity of the uniform temperature assumption, a heat transfer analysis was conducted on a 1 cm-thick ply of IM7/8552 based on experimental measurement of its exothermal heat during curing. The resulting maximum temperature gradient over a 40 μm distance, which corresponds to the characteristic size of the FE model evaluated in this work, was significantly lower than 1 °C. The assumption of uniform temperature is, therefore, acceptable.

During the curing process, the modeling region is assumed to be located inside a larger composite block with limited out-of-plane deformation due to high stiffness and low thermal and chemical expansion/shrinkage in the fiber direction. Accordingly, a symmetry boundary condition is imposed on model surfaces initially perpendicular to the fiber direction (Z-surfaces). Meanwhile, surfaces initially parallel to the fiber direction (X-surfaces and Y-surfaces) are left constraint-free due to the supposed lack of any external constraint in these directions. However, as the specimen is much larger in-plane compared to the model, a convergence study using models up to 16 times larger in the in-plane area was accomplished to verify the convergence of the residual stresses. The study successfully verified the convergence and applicability of the smaller models for this analysis. For more details on this convergence study, please refer to Appendix B.

Through the grinding process, Z-surfaces become constraint-free to replicate the machining, grinding, and specimen polishing processes, after which these surfaces become exposed and become the upper and lower surfaces of the membrane.

During curing and grinding, the model assumes no relative displacement at the fiber–matrix interface. Contact between fiber and matrix at the interface is modeled with Penalty Contact and No Separation in the normal direction and with Rough Friction in the tangential direction.

During the push-out process, X-surfaces and Y-surfaces are blocked, simulating the specimen fixed in the mount. The push-out process is simplified and modeled as two separate sub-steps: interface breaking and push-out. The interface breakage of the push-out fibers is modeled by modifying the contact properties into frictionless tangential contact interaction and allowing normal separation. Subsequently, fibers are pushed out by applying a displacement in the out-of-plane direction on their upper surface.

**Figure 7:**
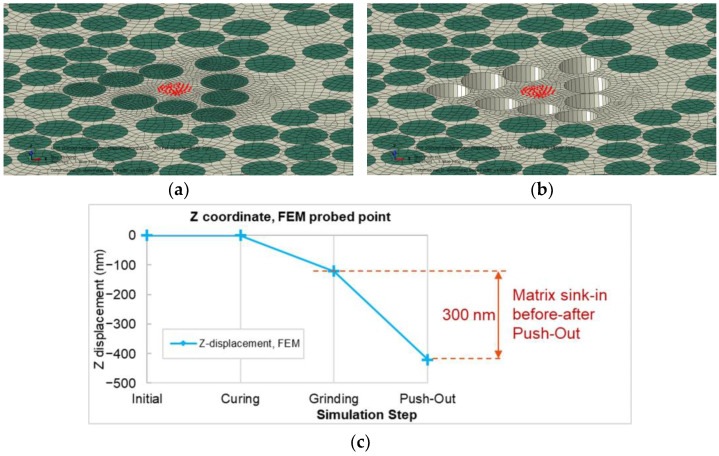
(**a**) FE model before push-out: red nodes corresponding to probed area. (**b**) FE model after push-out. (**c**) Z-displacement of probed area showing local matrix sink-in after push-out experiment.

In the FE model, the area probed by the nanoindenter is represented by a group of nodes (probed nodes, colored in red in Figure 7a,b) located at the corresponding position and within a circle of the same dimension as the nanoindenter tip (radius=2 μm). The Z-coordinate of this area is considered as that of the node with the highest vertical coordinate. Figure 7c shows an example of the vertical coordinate of the probed area during the simulation. The Z-coordinate remains constant during curing due to the symmetry boundary condition. After grinding, the matrix sinks in due to the removal of the vertical constraint and some amount of residual stress relief. Following fiber push-out, the matrix under tensile residual stress is released from the surrounding fibers’ constraints, leading to further sink-in that corresponds to the matrix displacement (sink-in) measured after the fiber push-out experiment.

### 4.3. Material Models and Properties

The curing behavior of the epoxy matrix is modeled using Abaqus built-in material curing model. For an epoxy material, the degree of cure (DOC) is commonly defined as the ratio $\alpha =Q/Q_{tot}$, where Q is the measured exothermal reaction heat and $Q_{tot}$ is the total exothermal heat, assuming the epoxy is fully cured. The Kamal equation is an established semi-empirical model that provides good fitting to experimental DOC data for epoxies [35,47,48]. In this paper, the evolution of DOC over time (or epoxy curing kinetics) is described using the Kamal equation in the form

(2) $$\dot{\alpha }=A_{1}\exp\left(-\frac{\Delta E_{1}}{RT}\right){\left(1-\alpha \right)}^{n}+A_{2}\exp\left(-\frac{\Delta E_{2}}{RT}\right)\alpha^{m}{\left(1-\alpha \right)}^{n},$$

where $\dot{\alpha }$ is the rate of cure;R and T are, respectively, gas constant and absolute temperature and $A_{1},A2,m,n,\Delta E_{1},\Delta E_{2}$ are effectively experimental fitting parameters. Using a simple linear transformation, Equation (2) can be directly implemented using Abaqus built-in curing kinetics. The set of material parameters ($m,n,A_{1},A2,\Delta E_{1},\Delta E_{2})$ is inversely determined using curve fitting with experimental DOC data, seeking a single value that produces the best fit to all experiments provided. The inverse/curve-fitting algorithm is implemented using Python with the function least_squares from the SciPy library. The algorithm is based on the Trust Region Reflective method.

**Figure 8:**
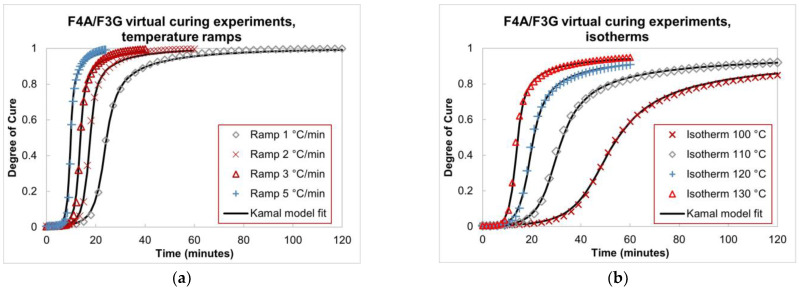
Data from virtual curing experiments and their fits using resulting Kamal equation model. (**a**) Cases with temperature ramps. (**b**) Cases with isotherms.

Due to the lack of curing data for the new F3G resin system, its cure kinetics is assumed to be similar to its base F4A resin system, both of which are cured at 121 °C. Schechter et al. [49] presented a curing model based on experimental DOC evolution of F4A subjected to eight different temperature profiles: four ramps, 1,2,3,5 °C/min; and four isothermal holds, between 100 °C and 130 °C. Virtual experimental data was generated for these eight curing temperature profiles using the curing model provided by Schechter et al. [49]. The data was used to find the Kamal equation parameters using inverse optimization. In this paper, the set of parameters in Table 1 is utilized to model F3G curing kinetics with Equation (2). Figure 8 presents DOC data from the eight virtual experiments and their fits with the Kamal curing model.

**Figure 9:**
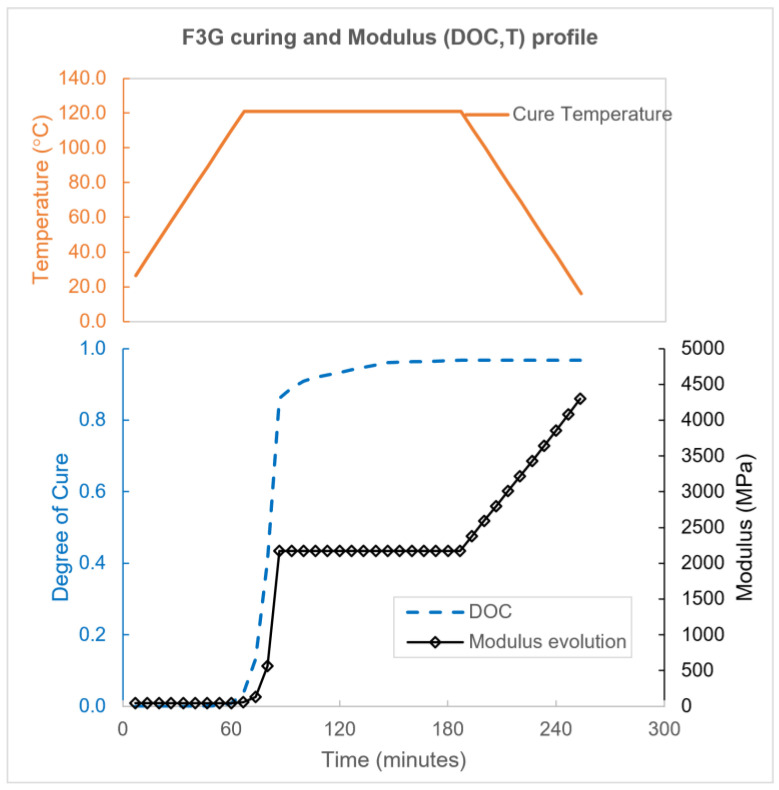
Cure cycle, DOC evolution, and modulus development for F3G epoxy.

The curing cycle provided by the F3G manufacturer is applied on the FE model, with a ramp rate of about 1.6 °C/min, a dwell at 121 °C for two hours, and a cooling of about −1.6 °C/min down to room temperature. The temperature profile and the DOC evolution of the epoxy are depicted in Figure 9.

The epoxy curing model also includes isotropic, linear chemical shrinkage $\varepsilon_{0}^{chem}$ that corresponds to fully cured epoxy (DOC=100%). For a given DOC, chemical shrinkage is proportional to the degree of cure: $\varepsilon^{chem}=\varepsilon_{0}^{chem}\times DOC$. In our FE model, the final DOC after the curing cycle is 96.7%.

F3G epoxy matrix is modeled as linear elastic, isotropic material. Table 2 provides the epoxy matrix properties at the end of its standard curing cycle. The epoxy modulus is measured by tensile testing. The epoxy CTE is provided by the manufacturer. Poisson ratio ν is estimated. The epoxy Young’s modulus varies during the curing simulation, while other properties are unchanged. Epoxy matrix modulus is dependent on both DOC and temperature and is provided to Abaqus in the form of a predefined table. Precise determination of modulus during curing as a function of DOC and temperature is difficult due to experimental constraints related to the strong coupling effects between the temperature, rate of chemical reaction, and resulting exothermal heat as the specimen is being cured [50]. In this paper, the modulus profile shown in Figure 9 is utilized for the FE epoxy model. The initial modulus equals 1% of the final modulus. It is worth noting that establishment of the chemical bond at the fiber–matrix interface is not modeled explicitly. Instead, a “smeared” or “phenomenological” approach is used where a tied-contact connection at the interface is defined throughout the analysis, and transmission of interfacial forces occurs through the modulus development model shown in Figure 9.

The F3G epoxy is modeled with the simplification of having no damage throughout the analysis. This is due to the lack of data on the new F3G material in addition to the lack of a model capable of describing the very complex dependence of epoxy damage properties in function of many parameters, including temperature [34,51], degree of cure [35], and humidity [34,51] which are also difficult to determine experimentally. Therefore, this simplification approach is taken, and the FEMU studies will be realized only on specimens where no visible matrix damage is observed during the experiment.

HS40 carbon fiber is modeled as a linear elastic, orthotropic material with thermal expansion. Its relevant parameters for the FE models are listed in Table 3. The longitudinal modulus value is from the datasheet [52]. Other parameters are estimated.

Thermal conductivity, mass density, and specific heat for both the fiber and the epoxy matrix are also required inputs for fully coupled thermal-stress analysis using Abaqus. However, due to the applied uniform temperature, these parameters have no effect on the FEM results.

Fibers and matrix are modeled by a mixture of C3D6T and C3D8T-type 3D elements.

## 5. Results and Discussion

### 5.1. FEMU Results and Application to Reproduce Matrix Sink-In

**Figure 10:**
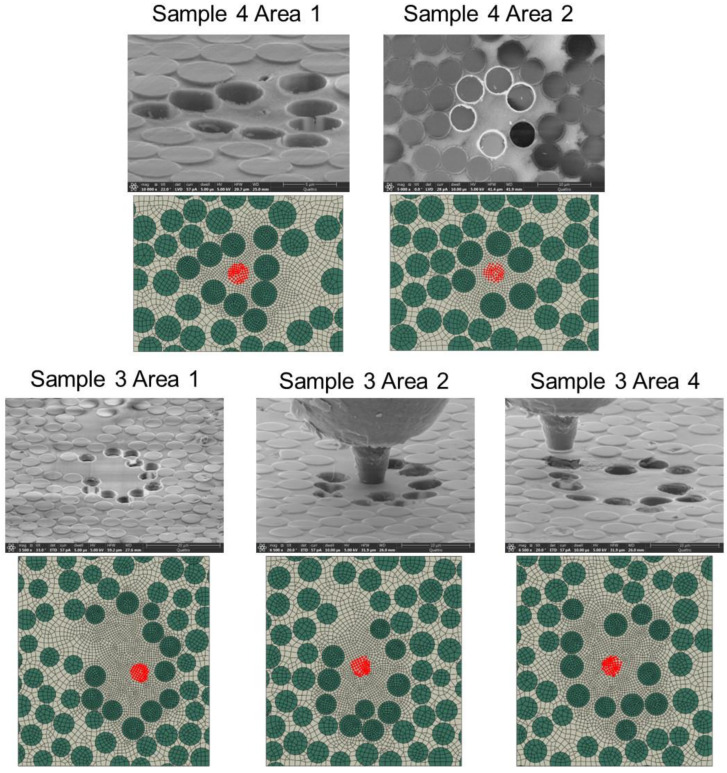
SEM images and FE geometries of experimental samples analyzed with FEMU.

The FEMU algorithm is applied to five different fiber push-out test areas corresponding to the five experiments. In this study, only areas without visible matrix damage, porosity defects, or any other imperfections visible in SEM data are selected for FEMU using a linear elastic matrix material model with no damage and representative of defect-free material. Figure 10 presents the SEM images and FE geometries of these five test areas. Table 4 presents results from FEMU studies. Chemical shrinkage values shown are the parametric $\varepsilon_{0}^{chem}$. Chemical shrinkage found by FEMU for the five selected experiments are consistent, showing a relatively small dispersion with a coefficient of variation of 17.1%. However, the total number of case studies is small. Validation of the method can be improved by including more experimental data.

**Figure 11:**
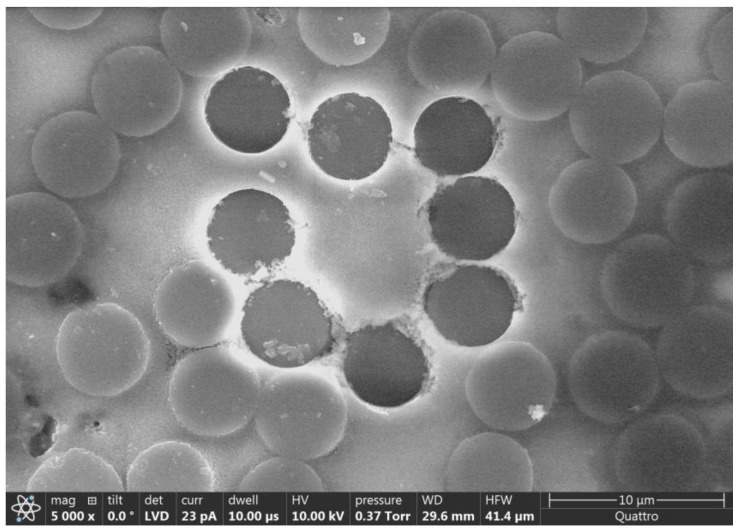
SEM image of Sample 5, Area 1, showing extensive matrix damage.

The average $\varepsilon_{0}^{chem}$ result from FEMU studies is then applied to the FE models of six other fiber push-out areas. Table 5 compares FEM sink-in prediction with experimentally observed results. The large difference between FEM and experimental data in the results shown in Table 5 was attributed to the presence of matrix damage in some of the experiments. Matrix damage was not accounted for in the simulation. For example, Sample 5, Area 3, and Sample 2, Area 3, showed little or no visible push-out damage, and the experimental sink-in measurements were close to FEM-predicted values. For test areas with visible matrix damage, including Sample 5, Area 1, Sample 2, Area 4, and Sample 2, Area 5, experimental sink-in was much higher than the FEM prediction. Figure 11 shows an example of significant matrix damage observed for Sample 5, Area 1. It is worth noting that experiments that resulted in visible matrix damage were discarded in the FEMU study presented previously, with results listed in Table 4.

Several additional factors are proposed as possible contributors to the large difference between FEM and experimental results. For instance, Ghaffari et al. [53] measured significant friction force at the broken fiber–matrix interface, in the order of 25% of the interface shear strength itself. Such a level of static friction stress at the interface might create and maintain significant residual stress in the nearby matrix, which is not considered in the current FE model of a free-sliding interface. Random variation of local material properties, such as voids or defects, cannot be ruled out, but it is hard to verify that experimentally. The local variation of matrix properties might be the reason for the experimental sink-in of Sample 5, Area 2 being much lower than the FE model estimation. Studying the influence of these factors requires additional experimental studies and improved FE modeling that includes these additional effects.

### 5.2. Evaluation of Residual Stress in Composite Using FE Modeling

On the FEM result, residual stress inside the referenced Sample 4, Area 1, after grinding and before push-out, is evaluated and discussed.

**Figure 12:**
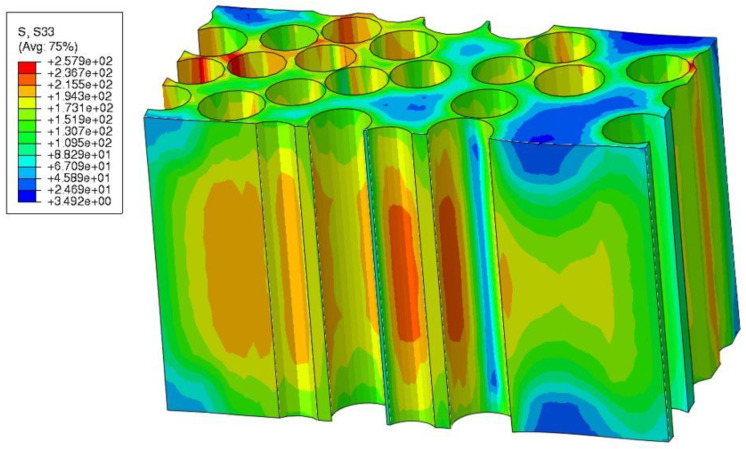
Before push-out: normal stress $S_{zz}$ in the fiber direction. Only matrix part is shown.

Figure 12 presents the matrix normal stress in the fiber direction ($S_{zz}$). Higher $S_{zz}$ values are observed in the areas close to the fibers, illustrating the fiber blocking that generates residual stress. Further away from the fibers into the matrix-rich areas, $S_{zz}$ is lower thanks to the partial residual stress release and smaller blocking effect from the fibers. Normal stress values are very high compared to F3G tension strength provided by the manufacturer (74.6 MPa).

**Figure 13:**
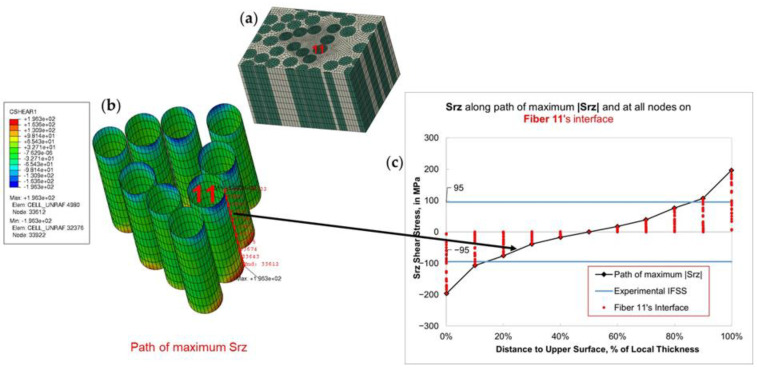
After grinding: (**a**) maximum shear $S_{rz}$ is located on Fiber ID 11; (**b**) path of maximum shear $S_{rz}$ runs through local membrane thickness; (**c**) shear stress $S_{rz}$ plotted for all nodes on the interface of Fiber ID 11, along with average experimental *IFSS* for HS40/F3G carbon/epoxy interface.

**Figure 14:**
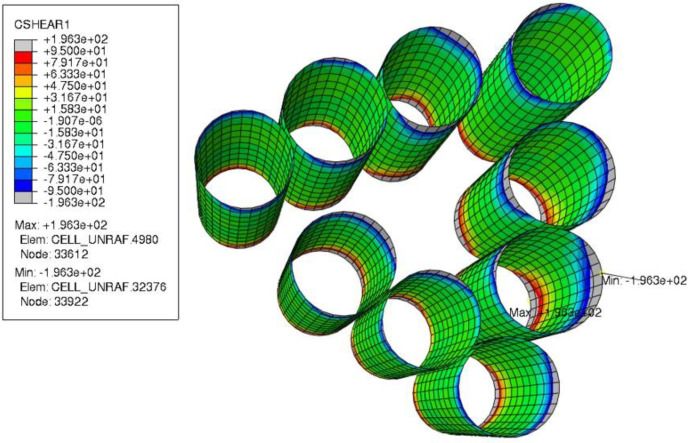
Before push-out: shear stress $S_{rz}$ at the interfaces of all push-out fibers. Areas where $\left|S_{rz}\right|>IFSS$ are colored gray.

On the interface of push-out fibers, interfacial shear stress in the fiber axial direction ($S_{rz}$) is presented in Figure 13. $S_{rz}$ has the same orientation as the *IFSS* that is measured by the fiber push-out test. Mesh convergence has been verified so that maximum $S_{rz}$ in the model is produced at the same location, which is on the interface of fiber with the ID 11 (Figure 13a) that faces outside into a matrix-rich area (Figure 13b). In Figure 13c, shear stress $S_{rz}$ is plotted for all nodes on the interface of Fiber 11 against their relative through-thickness coordinates. A “path of maximum $S_{rz}$” is highlighted, showing the through-thickness path that crosses the point of maximum $S_{rz}$. Experimentally measured shear strength of HS40/F3G interface (IFSS=95 MPa, as reported by Ghaffari et al. [25]) is also plotted in both shear directions for comparison. The stress distribution is symmetric, reflecting the through-thickness symmetry of the current FE model. Shear stress magnitude is zero at the midpoint and increases when approaching the membrane surfaces, eventually surpassing interfacial strength (*IFSS*) in many areas. The high stress developed in the FE model is further illustrated in Figure 14, where interface areas with shear stress $S_{rz}$ higher than shear strength IFSS are colored gray. Large interface areas with $S_{rz}>IFSS$ are also observed on FEM results of other push-out areas.

**Figure 15:**
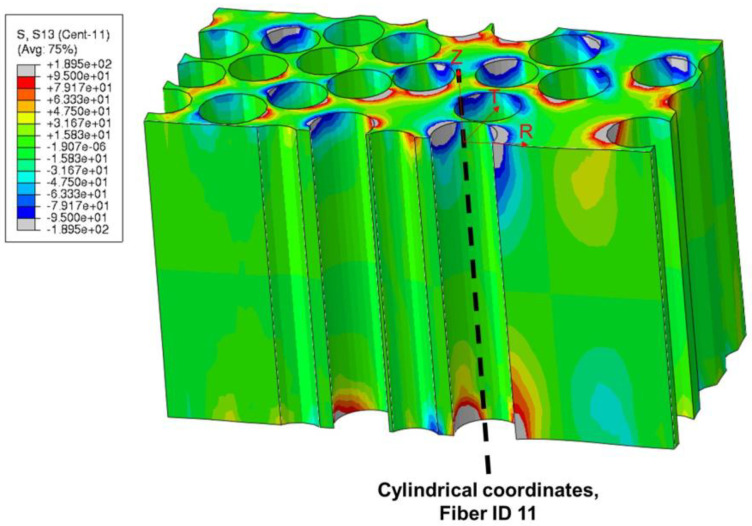
Before push-out: shear stress $S_{rz}$ in the matrix in cylindrical coordinate system at fiber ID 11 axis. Areas where $\left|S_{rz}\right|>IFSS$ are colored gray.

Figure 15 presents the radial–axial shear stress $S_{rz}$ in the matrix in a cylindrical coordinate system located at the central axis of Fiber ID 11 by showing a cut through the FE model. Areas where matrix shear stress $S_{rz}>IFSS$ are also colored gray to better emphasize the very high level of shear stress and their concentration near the fiber–matrix interface at the membrane surfaces. The result illustrates the transfer of fiber interfacial shear stress into the matrix. Near the fiber, matrix shear stress closely corresponds to interfacial shear stress. Further from the fiber and into the matrix-rich area, matrix shear stress becomes lower.

These evaluations of residual stress suggest possible premature failure at the fiber–matrix interface and possible matrix damage before the fiber push-out experiment. However, on the SEM image, no obvious interfacial damage can be observed on the membrane surface (Example: Figure 2a showing Sample 4, Area 1 before push-out). One possible reason is that the simplification of the linear elastic matrix makes the model too stiff, which leads to generating very high stress. The same simplification might also lead to different stress distribution compared to a model with elasto-plastic matrix. For instance, epoxy matrix might yield in shear, reducing the shear stress suffered by the interface to below the interface shear strength, thus causing no visible interface damage. Further studies with improved matrix material models are being conducted to investigate these possibilities.

### 5.3. Limitations and Potential Improvements

Due to the difficulty of conducting microscale experiments and the corresponding lack of data, many material and model properties have to be assumed or simplified. These assumptions and simplifications likely have significant effects on the FEM simulation of these phenomena considering the strong and complex interaction between different component materials in the composite. Improvement of material models would increase the fidelity of the method. For instance, better determination of fiber properties, especially in the transverse direction, is desired since the fiber is much less stiff in this direction, so the interaction with the surrounding matrix might be more significant and complex, including effects on the interfacial friction. Though still smaller than the maximum values reported [29], epoxy matrix chemical shrinkage found by studies presented in this paper of 3.7% on average (at 96.7% DOC) is higher than common experimental measurements of chemical shrinkage for macroscale epoxies (typically 2% to 3% [27,28,29]). This comparison adds to the importance of further investigations and improvements to the proposed method.

Regarding the epoxy curing model, better determination of cure temperature (thick plies might exhibit significant temperature difference at the core due to exothermal heat) might also increase the precision of the curing history, especially considering the rate of the cure being very sensitive to temperature at a certain range. Viscoelasticity and resin flows during curing should also be considered since the curing duration is relatively long, which might lead to some spontaneous stress relaxation within the specimen.

At the current stage, the analysis technique requires that the fibers are pushed out without damaging the matrix, so special care must be taken during a fiber push-out experiment.

Assumptions and simplifications were made during the study of specimen geometries and their reproduction in FE models, such as no image distortion, perfectly circular fiber cross-section, and all fiber axes perfectly perpendicular to the membrane plane (i.e., no fiber misorientation or misalignment). Errors due to these assumptions and simplifications have not been characterized.

Boundary conditions representing the specimen in the fixture during the fiber push-out experiment are also simplified, assuming no in-plane stress is introduced due to the taping on the sides of the membranes. An alternate approach is to perform the fiber push-out experiment with the “cave configuration” proposed by Ghaffari et al. [25] to eliminate uncertainty in the specimen boundary conditions likely affecting the stress field in the membrane.

## 6. Concluding Remarks

This paper presents a new methodology capable of assessing residual microstresses in CFRPs using fiber push-out experiments with in situ SEM and data-driven FEMU. The method is illustrated for the evaluation of residual stress in a carbon-fiber/epoxy–matrix HS40/F3G composite material system. It is worth noting that very high process-induced residual microstresses are predicted, which may result from possible artifacts and inconsistencies in the FEMU procedure. However, these preliminary results are worthy of the attention of the materials community as they emphasize the following key findings. (A) The residual microstresses are, indeed, unexpectedly high, as shown by the in situ experiments measuring an average out-of-plane matrix deformation associated with the release of residual stresses larger than 1% of the specimen thickness in the region of the pushed-out fibers. (B) The importance of the in situ data-driven analysis methods cannot be underemphasized. Such methods can capture complex physics phenomena at appropriate scales and enable an improved understanding of material response essential to integrated computational materials engineering and material design. This work presents an initial reference point in the quest for higher fidelity assessment of the process-induced residual microstresses. There is a lot of room for improvement, such as increasing the accuracy of the boundary conditions and material properties, including matrix inelastic properties and fiber–matrix interface properties, among others. Moreover, in situ, SEM-based full-field measurements of the deformation and 3D surface shape after fiber push-out may improve the fidelity of the residual microstress assessment.

## Figures and Tables

**Table 1:** Kamal curing kinetics constants for F4A/F3G epoxy resin from fitting to virtual test data.

Kinetics Parameter	Value	Unit
m	1.29	-
n	2.70	-
$A_{1}$	$2.32\times 10^{15}$	$s^{-1}$
$A_{2}$	$3.20\times 10^{7}$	$s^{-1}$
$\Delta E_{1}$	$1.50\times 10^{5}$	J/mol
$\Delta E_{2}$	$6.99\times 10^{4}$	J/mol

**Table 2:** F3G epoxy matrix material properties, after-cure.

Elasticity	E (MPa)	ν
	4300	0.39
Thermal properties	$CTE\;(ppm{/}^{o}C)$	
	42.7	
Curing properties	$\varepsilon_{0}^{chem}$	
	variable	

**Table 3:** HS40 carbon fiber material properties.

Elasticity	$E_{11}\;(GPa)$	$E_{22}\;(GPa)$	$E_{33}\;(GPa)$	$\nu_{12}$	$\nu_{13}$	$\nu_{23}$	$G_{12}\;(GPa)$	$G_{13}\;(GPa)$	$G_{23}\;(GPa)$
	455	13	13	0.3	0.3	0.46	11.3	11.3	44.5
Thermal properties	$CTE\;(ppm{/}^{o}C)$								
	−0.7								

**Table 4:** FEMU results on 5 selected fiber push-out areas with no visible matrix damage.

Test Area	Experimental Sink-In (nm)	Chemical Shrinkage ($\varepsilon_{0}^{chem}$, 100% DOC)
Sample 3 Area 4	360	4.91%
Sample 3 Area 2	328	4.10%
Sample 3 Area 1	319	3.68%
Sample 4 Area 2	260	3.24%
Sample 4 Area 1	300	3.43%
	**Mean**	**3.87%**
	Standard Deviation	0.66%
	**Coefficient of Variation**	**17.1%**

**Table 5:** Matrix sink-in for other test areas with FEMU result application.

Test Area	Visible Matrix Damage?	FEM-Predicted Sink-In (nm)	Experimental Sink-In (nm)	Difference
Sample 5, Area 1	Yes	257	530	106%
**Sample 5, Area 2**	**No**	**258**	**144**	**−44%**
**Sample 5, Area 3**	**No**	**178**	**131**	**−26%**
**Sample 2, Area 3**	**No**	**299**	**351**	**17%**
Sample 2, Area 4, p1	Yes	120	390	225%
Sample 2, Area 4, p2	Yes	152	340	124%
Sample 2, Area 5	Yes	62	345	456%

**Figure A1:**
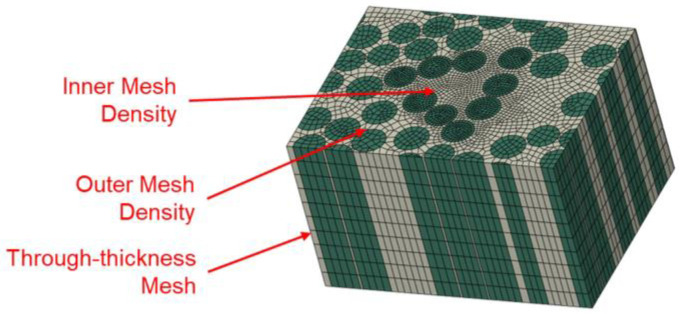
Mesh parameters of the FE Model.

**Figure A2:**
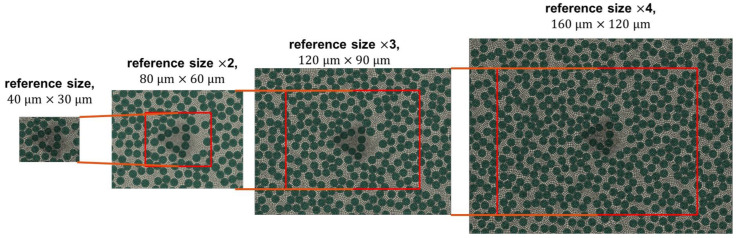
Different model sizes for convergence study. Smaller model is the “central part” of a larger model.

**Figure A3:**
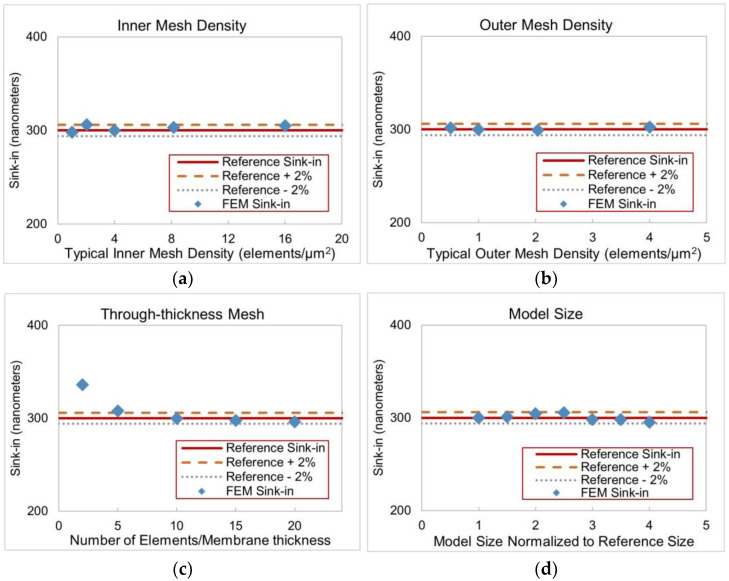
Convergence study results: (**a**) inner mesh density; (**b**) outer mesh density; (**c**) through-thickness mesh; (**d**) model size.

**Figure A4:**
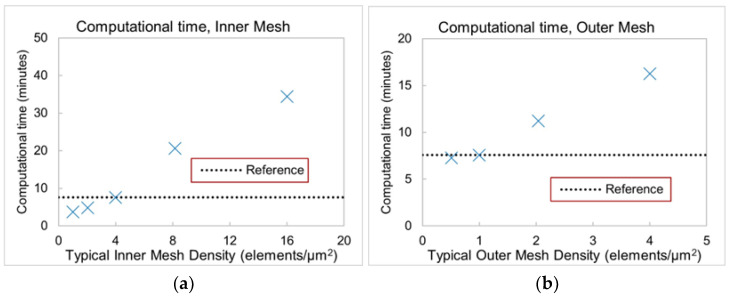

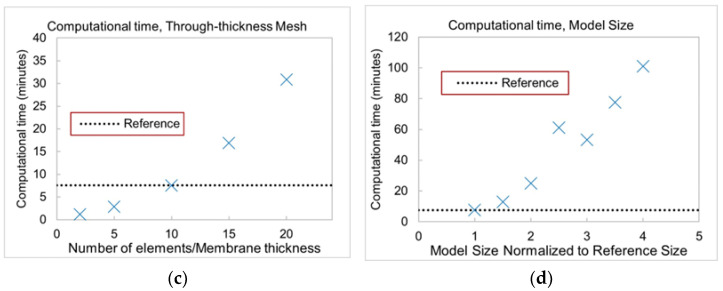
Computational time for the convergence studies: (**a**) inner mesh density; (**b**) outer mesh density; (**c**) through-thickness mesh; (**d**) model size.

**Figure A5:**
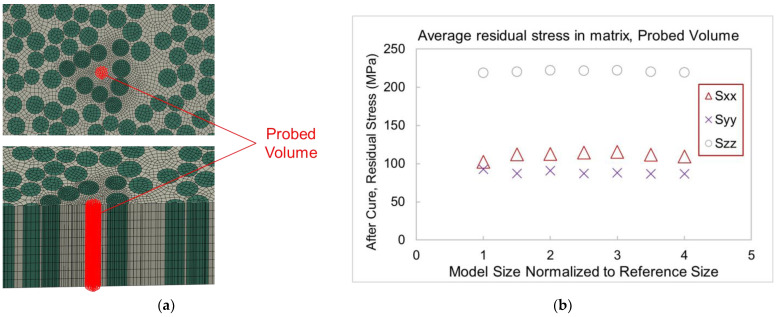

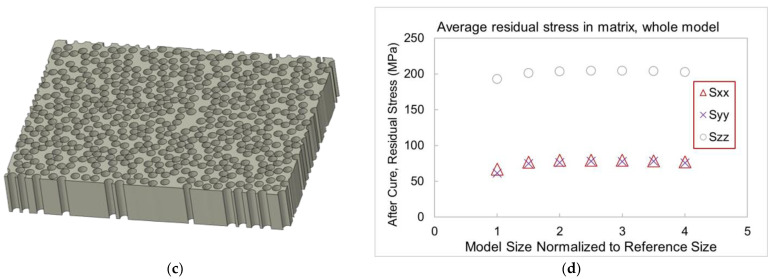
(**a**) Probed volume: the matrix volume under the probed area. (**b**) Average residual stress in probed volume at different model sizes. (**c**) The whole matrix volume in FE model. (**d**) Average residual stress in the whole matrix volume at different model sizes.

**Figure A6:**
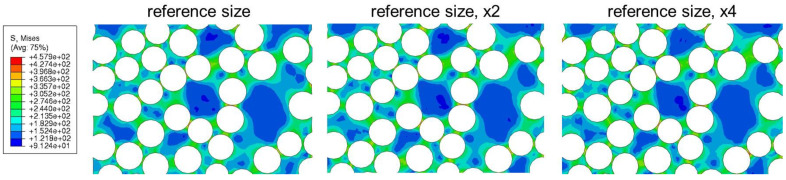
After curing: on an in-plane cut through the middle of membrane thickness, von Mises stress in matrix is shown at fiber push-out area for different model sizes.

## Data Availability

Not applicable.

## Appendix A

For mesh and size convergence studies, models assume reference geometry (Figure 5) for the inner area. Reference size has in-plane dimensions of 40 μm×30 μm. Convergence study utilizes the measurement of post-push-out matrix sink-in.

FE model’s meshing is described by three parameters (Figure A1): inner mesh density around push-out fibers; outer mesh density around the rest; and through-thickness mesh as the number of elements along the membrane’s out-of-plane dimension. In-plane mesh density is higher around the push-out fibers than the rest of the model to effectively capture deformation in the interested area. Mesh convergence is assumed when variation in matrix sink-in is less than 2% with a double mesh density increase. From mesh convergence analyses (Figure A3a–c), the following reference mesh configuration satisfies the assumed convergence criterion: inner mesh density at four elements per $\mu m^{2}$; outer mesh density at one element per $\mu m^{2}$; through-thickness mesh at 10 elements along the thickness.

For the size convergence study, each smaller-sized model’s geometry is a rectangle “central portion” of the maximum-sized model, as depicted in Figure A2. The size convergence study result presented in Figure A3d suggests that the current FE model converges at a relatively small reference size, which contains the push-out fibers and a surrounding extension of several fiber diameters in the in-plane directions.

The computational time for convergence studies is presented in Figure A4. FE simulations are performed using 11 CPUs of the Intel Xeon Processor X5650. The simulation of the reference FE model takes approximately 8 min, while the simulation for the largest FE model of four times the reference size takes approximately 100 min.

## Appendix B

To verify the validity of the application of the far-field free in-plane boundary conditions (BCs) to the microscale FE model for the curing simulation, a convergence study is performed. For models with different in-plane dimensions, residual stress generated at the end of curing is analyzed. The analysis studies average stresses in the probed volume (Figure A5a), which includes the matrix volume elements under the probed area, and in the whole matrix volume of the FE model (Figure A5c). Figure A5b presents the volume average in-plane stresses Sxx,Syy and the through-thickness stress Szz at the probed volume. Figure A5d presents Sxx,Syy and Szz in the whole matrix volume of the FE model. Figure A6 presents the von Mises stress at the fiber push-out area for different model sizes on an in-plane cut through the middle of membrane thickness, allowing visualization of residual stress distribution in this area.

Convergence in model size is observed for the residual stress both at the local probed volume and in the whole matrix volume of the model. Von Mises stress distribution in the fiber push-out area also shows strong similarities between models of different sizes. These results suggest that applying the current in-plane BCs to the FE model would generate similar local residual stress regardless of the model size. Therefore, the application of the far-field in-plane BCs of macroscale specimen to the microscale FE model is valid for the purpose of residual stress analysis.
